# Classification of Non-Severe Traumatic Brain Injury from Resting-State EEG Signal Using LSTM Network with ECOC-SVM

**DOI:** 10.3390/s20185234

**Published:** 2020-09-14

**Authors:** Chi Qin Lai, Haidi Ibrahim, Aini Ismafairus Abd Hamid, Jafri Malin Abdullah

**Affiliations:** 1 School of Electrical and Electronic Engineering, Engineering Campus, Universiti Sains Malaysia, Nibong Tebal 14300, Penang, Malaysia; chiqinlai@student.usm.my; 2 Brain and Behaviour Cluster, Department of Neurosciences, School of Medical Sciences, Universiti Sains Malaysia, Health Campus, Jalan Raja Perempuan Zainab 2, Kubang Kerian 16150, Kota Bharu, Kelantan, Malaysia; aini_ismafairus@usm.my (A.I.A.H.); jafrimalin@usm.my (J.M.A.)

**Keywords:** deep-learning, electroencephalogram, error-correcting output coding, long short term memory network, machine-learning, resting-state, support vector machine, traumatic brain injury

## Abstract

Traumatic brain injury (TBI) is one of the common injuries when the human head receives an impact due to an accident or fall and is one of the most frequently submitted insurance claims. However, it is often always misused when individuals attempt an insurance fraud claim by providing false medical conditions. Therefore, there is a need for an instant brain condition classification system. This study presents a novel classification architecture that can classify non-severe TBI patients and healthy subjects employing resting-state electroencephalogram (EEG) as the input, solving the immobility issue of the computed tomography (CT) scan and magnetic resonance imaging (MRI). The proposed architecture makes use of long short term memory (LSTM) and error-correcting output coding support vector machine (ECOC-SVM) to perform multiclass classification. The pre-processed EEG time series are supplied to the network by each time step, where important information from the previous time step will be remembered by the LSTM cell. Activations from the LSTM cell is used to train an ECOC-SVM. The temporal advantages of the EEG were amplified and able to achieve a classification accuracy of 100%. The proposed method was compared to existing works in the literature, and it is shown that the proposed method is superior in terms of classification accuracy, sensitivity, specificity, and precision.

## 1. Introduction

Health care insurance is a policy that covers a part or all of an individual’s risk of incurring medical costs when there has been illness, injury, or trauma. Nevertheless, several individuals commit health care fraud by presenting a false diagnosis of illnesses. Health care fraud, according to the National Health Care Anti-Fraud Association, is deliberate deceit or false representation rendered by an individual or organization that can lead to some undue benefit for him or his accomplices [1]. Thus, health care insurance fraud has caused a worldwide loss of tens of billions of dollars annually and poses a critical problem for the insurance businesses [2]. The identification of fraud in health care thus plays a crucial role in preventing these scenarios.

Traumatic brain injury (TBI) happens at a high rate, with more than 50 million cases per year worldwide [3]. In conjunction, TBI occupied a large portion of the health care insurance claims. Medical images of the brain from the hospital and analysis reports by professionals would be provided to the insurance companies to evaluate the brain injury claims. The golden standard of medical imaging for TBI is the computed tomography (CT) or magnetic resonance imaging (MRI) [4]. Even then, conducting a CT or MRI scan for any patient who demanded medical claims is restricted due to limited resources in hospitals [5]. The inquiry also required a considerable workload to verify or deny the allegations made, which is a time-consuming process for human experts.

TBI can be divided into three levels of severity; severe TBI, moderate TBI, and mild TBI. Insurance claims for the severe TBI are easier to justify because the severe TBI patients often have a period of unconsciousness [6]. On the other hand, identifying the mild TBI and moderate TBI patients without medical imaging analysis posses a challenge. The mild TBI is the least severe among the trauma, and biomarkers of the mild TBI’s pathophysiologic effects were not established for clinical use. Neuroimaging technologies are thus required to provide a compelling rationale for mild TBI. Similarly, post mild TBI patients often experience acute short-term symptoms, for example, loss of focus, memory loss, headache, sensitivity to light, fatigue, and irritability [7]. Some of those symptoms, however, healed within two to three weeks. Therefore, neuroimaging, such as a CT scan, needs to be performed to validate mild TBI insurance claims.

Justification of moderate traumatic brain injury (TBI) remains a problem because its severity is impermanent. Patients with moderate TBI can experience an acute phase period in which both intra-cranial and inter-cranial traumas may cause secondary brain injury, increasing the severity of TBI [8]. Contrary to this, one study has found that patients with moderate TBI did less well. Patients demonstrate a good recovery at approximately 60% [9]. Therefore, conventional medical imaging, such as CT or MRI, must be done to assess a moderate TBI patient’s actual health status. Yet, to promptly perform neuroimaging for each submitted claim of mild and moderate TBI patients is not feasible due to limited hospital resources. It is also costly to conduct CT or MRI scanning [10]. Therefore, a high workforce effort is required to support the medical statements made and neuroimaging findings.

Recent advances have shown that electroencephalogram (EEG) is a prospective modality for the instant detection of TBI. Studies have shown that biomarkers can be identified by analyzing the frequency band of quantitative EEG (qEEG), which are the alpha, beta, theta, and gamma bands. It was found that reduction in the mean value of the alpha frequency band and the increase of the theta band activities as compared to a healthy person is related to TBI [11,12,13,14].

The analysis of qEEG manually is tedious and requires a lot of human resources, as the recorded EEG is usually long in duration and uses multi-channels. Hence, there is a need for an automated system to perform the analysis. Multiple surveys and studies have been done, and they provide an insight for usability and the future of utilizing deep learning in analyzing biomedical data [15,16,17,18,19], enlightening that deep learning works well to perform prediction and analysis using biomedical data. Therefore, machine learning approaches were used frequently in the literature to enable automated identification of TBI. A decent review has been done by Rapp et al. [20] in supporting the usage of EEG in TBI identification.

Two types of EEG used for TBI identification are active paradigms EEG and task-free paradigms EEG. During the recording of active paradigms EEG, subjects must perform certain tasks or are exposed to external stimulants [21,22]. To detect TBI, McBride et al. [23] implemented active paradigms EEG that required the subject to do memory tasks during EEG recording. They used a support vector machine (SVM) with features taken from event-related Tsallis entropies. Their experimental results indicated that EEG is a promising tool for early screening of TBI. Fisher et al. [24] have proposed a method that can track neural electrophysiological abnormalities following head injury in real-time, by using cortical somatosensory evoked electroencephalographic potentials (SSEPs) on an animal model. A significant increment in EEG entropy and alterations in low-frequency components have been found concerning TBI. Active paradigms EEG are usually recorded to assess the functionality and responses of the human brain post injured [25].

Another TBI classification study based on animal models can be found in work by Vishwanath et al. [26]. Their proposed method explored multiple classifiers, including decision trees (DT), random forest (RF), neural network (NN), SVM, K-nearest neighbors (KNN), and convolutional neural network (CNN). These classifiers were analyzed based on their performance in classifying mild TBI (mTBI) data. Average power in various frequency sub-bands and alpha to theta power ratio in animal model EEG were extracted as input features for machine learning approaches. Results from their study suggested similar procedures are applicable to detect TBI in humans in practical scenarios.

In addition to the existing work using active paradigms EEG, Cao et al. proposed an automatic classification of athletes with a concussion using an EEG-based SVM [27]. Their approach can detect mild TBI in athletes and determine whether they are suitable to return-to-play (RTP) or not. A Fast Fourier Transform (FFT) is performed on the pre-processed signal, and the signal was divided into theta, alpha, beta1, beta2, and beta3. Average powers were calculated for each of the frequency bands. In addition to the feature set, average powers for individual 1 Hz frequency components between 1 and 30 Hz for all the electrodes were computed. Feature reduction was performed to reduce the number of features, using heuristic minimal redundancy maximal relevance (MRMR) framework. The features were ranked based on mutual information. The top 10 features were selected and directed to an SVM to classify the healthy subject and mild TBI patient.

In the work of Thornton et al. [28], qEEG features have proven to be useful in the diagnosis and rehabilitation of the cognitive problems of the traumatic brain injured (TBI) subject. Their work extracted relative power, spectral correlation coefficient, and phase different from active paradigms EEG, where the subjects were required to perform a set of cognitive tasks during the recording. Subsequently, discriminant analysis was carried out based on the features to differentiate between mild TBI subjects and healthy controls.

For the recording of task-free paradigms EEG, subjects are not required to perform the task or being exposed to external stimulants. Task-free paradigms can be divided into eyes close and eyes open EEG. McNerney et al. [29] developed a mild TBI detection using adaptive boosting (AdaBoost) with resting-state EEG as its input. The resting-state EEG undergone steps of pre-processing to eliminate unwanted substances in the signal. A bandpass filter with cutoff frequency from 0.1 Hz to 100 Hz was first applied to the raw EEG. Next, artifacts and spikes were manually labeled and removed. Subsequently, power spectral densities (PSD) of the alpha, theta, delta, and gamma bands were computed from AF8 to FpZ and AF7 to FpZ of the cleaned signal. The mean PSD logarithm for every channel for respective frequency bands was obtained and concatenated into a feature vector. The AdaBoost classifier was trained by using the feature vector, and their results achieve high classification accuracy.

Also, Brink et al. [30] presented a task-free based EEG approach that makes used of the Naive Bayes classifier to detect severe TBI. A notch filter was applied to the raw EEG to remove the electrical line noises. A low pass filter was subsequently implemented to the resulting signal with a 0.5 Hz cutoff frequency. Similarly, the artifacts were removed manually using visual inspection. The cleaned EEG of each subject was segmented into two-seconds segments. The relation between the log-transformed orthogonalized amplitude from three frequency bands, which are the theta, delta, and gamma bands, is determined [30]. Their approach can detect severe TBI and has shown good detection accuracy.

A study has been carried out by O’Neil et al. [31] using resting-state EEG, which generates a TBI index to classify positive CT scan subjects and negative CT scan subjects. In their work, qEEG features of absolute and relative power, mean frequency, inter- and intra-hemispheric coherence, and symmetry computed for the delta, theta, alpha, beta, and gamma frequency bands. A binary discriminant classification algorithm was developed based on the extracted features to generate the TBI index for classification purposes.

By developing a sequence of binary classifiers, Prichep el al. [32] developed an approach that can perform multiclass classification (i.e., classifying normal control, concussed subject, and structural injured/ CT scan positive subjects). Their method extracted age-regressed quantitative features (linear and nonlinear) resting-state EEG signals. Extracted features underwent a unique data reduction method before directed to the classifiers to maximize confidence of prospective validation and minimizing over-fitting.

On the other hand, Prichep el al. [33] evaluated three different classifiers (i.e., Ensemble Harmony, Least Absolute Shrinkage and Selection Operator (LASSO), and Genetic Algorithm (GA)) using absolute and relative power, mean frequency, inter- and intra-hemispheric coherence and symmetry computed for the delta, theta, alpha, beta and gamma frequency bands extracted from resting-state EEG. Their proposed method classified CT scan positive patients from CT scan negative patients. Hanley et al. [34] proposed a brain structural injury classifier (i.e., classifying CT positive and CT negative patients) based on a binary discriminant classification algorithm, which was derived using a Least Absolute Shrinkage and Selection Operator methodology. Power, phase, coherence were extracted from the resting-state EEG as input features to the classifier. To determine quantitative resting-state EEG biomarkers for mild TBI, Lewine et al. [35] utilized multiples classifiers to investigate the useful measures to identify and classify mild TBI. Quantitative metrics included absolute and relative power in delta, theta, alpha, beta, high beta, and gamma bands, plus a measure of interhemispheric coherence in each band. Mentioned quantitative metrics were used as an input to the respective classifiers.

Although active paradigms have shown promising results in detecting TBI, it requires extensive setup time for the EEG recording. Some of the active paradigms require patients to have higher cognitive capability and attention. The necessity of active paradigms is to assess the sensory pathways functionality and responses of the human brain post-injury [25]. Accordingly, task-free paradigms do not require patients to respond to stimulants’ tasks, making it a better option for the TBI detection approach. During the acute phase period, moderate TBI patients can be in coma states and cannot complete a task or react to the stimulant provided. Task-free paradigms have the benefit of not interrupting the sleep cycle of patients [36,37].

Machine learning approaches to detect TBI require appropriate feature extraction and selection to achieve better detection accuracy. It can also be highlighted that the state-of-art approaches do not consider the signal’s temporal dependency, although EEG is high in temporal resolution. Researchers have been putting efforts and have proposed a long short-term memory (LSTM) networks, which can address the long term temporal dependence issue. LSTM is a subclass of recurrent neural networks (RNN) first suggested by Hochreiter and Schmidhuber [38] then modified by Graves [39].

Some researches have explored the use of LSTM on non-medical EEG-based applications. Most of the EEG-based LSTM applications were used in brain-computer interface (BCI), such as motor imagery classification [40,41,42,43,44,45,46], emotion classification [47,48,49,50,51,52], depression detection [53,54,55], biometrics [56,57], sleep stage classification [58,59,60,61,62,63], driving behavioral classification [64,65], directional signal classification [66], machine health monitoring [67] and EEG signal classification [68]. There are some research works on LSTM for medical applications reported in the literature [69,70,71,72,73,74,75], but as far as our concern, there is still no approach being proposed to identify TBI using LSTM networks.

Although the LSTM network can magnify EEG’s temporal advantages, no attention is given to the development of non-severe TBI classification from the literature. LSTM is an improved recurrent neural network (RNN) that overcome the shortage of failing to learn in the presence of time lags larger than five to 10 discrete time steps between relevant input events and target signals [76]. In contrast with RNN, LSTM contained cells that select important information to retain and unrelated information to be released. Therefore, LSTM carries potential that can learn one time step at a time from all 63 EEG channels, rather than an overall feature extraction. It is believed that retaining important information from the previous time step stores correlation information from the EEG time series through time and enables more quality architecture learning.

This paper presents an EEG-based LSTM with error-correcting output coding SVM (LSTM ECOC-SVM) architecture that can classify non-severe (i.e., mild and moderate TBI) from healthy subjects. From the literature, analysis and prediction of TBI from EEG using conventional computational intelligence approaches are tedious as they usually involve complicated feature extraction or feature selection of the signal. This study contributes to the body of knowledge by presenting an architecture that does not require extensive feature extraction and feature selection from the EEG signal compared to existing literature works, yet provides high classification performance. This paper consists of four main sections. Section 1 is an introduction to this study, including some background and literature reviews. The subsequent section (i.e., Section 2) presents the dataset and an overview of the proposed architecture. The later part of the section discussed the training procedure and performance measures used in this study. In Section 3, experiments that are conducted to design the proposed architecture are presented. Further, in this section, the results are also presented together with detailed discussion and analysis. The final section (i.e., Section 4) summarizes the output from the conducted experiments, proposed architecture, and its performance.

## 2. Materials and Methods

### 2.1. Data Acquisition

All 36 resting-state eyes-closed EEG recordings utilized in this research were obtained from the Hospital Universiti Sains Malaysia, Kelantan, Malaysia, under ethical clearance USM/JEPeM/15110486. These EEG recordings were contributed by 36 volunteers, with whom 12 of them suffered from mild TBI, another 12 of them suffered from moderate TBI, and the remaining 12 persons are healthy individuals. The age range of all of the subjects is between 18 to 65 years old. All TBI patients sustained nonsurgical mild TBI (i.e., GCS score between 9 to 12) or moderate TBI (i.e., GCS score between 14 to 15). They endured the initial hit involving the left frontal-temporal-parietal lobe, which was confirmed by a CT scan. Every volunteer is asked to close his/her eyes during data acquisition to get the eyes-closed resting-state EEG records. There are no tasks or activities performed during the data acquisition (i.e., task-free EEG recording).

The EEG signals were acquired by utilizing 64 electrodes, arranged using the international 10-10 EEG electrode practice to record the brain’s electrical signals from 64-sites on the scalp. WaveGuard EEG cap is used to mount these electrodes. In this research, $CP_{z}$ channel is excluded because it is taken as the Electrooculography (EOG) channel. Thus, there are only 63 EEG functional channels used for the input data in our classification approach. The electrodes’ impedance is set to be below 5kOhm with the connected earlobes serving as the reference, and the ground electrode is positioned 10% anterior to Fz. A programmable DC-coupled broadband SynAmps amplifier (accuracy of 0.033/bit, and gain of 2500) is employed to record the EEG signals. The recording range is set to ±55 mV at the frequency range from DC to 70-Hz. The digital EEG signals are obtained by utilizing a sampling frequency $F_{s}$ of 1000 Hz and using 16-bit analog-to-digital converters. The digital EEG signal *d* of channel *i* at discrete data point *n*, which is $d_{i}\left[n\right]$, is obtained from the analog EEG signal *a* at the corresponding channel. This digital signal can be defined as [77]:

(1) $$d_{i}\left[n\right]=a_{i}\left(nT\right)=a_{i}\left(\frac{n}{F_{s}}\right).$$

The conversion of the analog EEG signal to the coresponding digital EEG signal took place by taking samples (i.e., sampling) at each sampling time interval, *T*, of the analog EEG signal [77]. In this work, the value of *T* (i.e., $1/F_{s}$) is one millisecond.

### 2.2. Data Preparation and Pre-Processing

The recorded EEG signals were pre-processed to eliminate unwanted elements, which will affect the training of the proposed architecture (i.e., artifacts and electrical line noises). Firstly, the EEG is filtered with a 50 Hz notch filter to remove electrical lines from the EEG as the electrical line frequency in Malaysia is 50 Hz. Next, the resultant signal has to undergo a bandpass filter of 0.1 Hz and 100 Hz. It was suggested that the frequency analysis of TBI is limited to a frequency band between 0.1 Hz and 100 Hz, which is then further divided into several sub-bands (i.e., delta, theta, beta, alpha, and gamma bands) [20]. From the literature, it can also found that a bandpass filter of 0.1 Hz and 100 Hz is commonly used in work related to TBI [29]. As physiology is best understood for these frequency bands, using a bandpass filter of 0.1 Hz and 100 Hz enables the analysis of TBI to be carried out focusing on the delta, theta, beta, alpha, and gamma bands.

Subsequently, the signal is downsampled from 1000 Hz to 100 Hz (i.e., using a downsampling integer factor *D* of 10). Downsampling is commonly used in the EEG processing task as it can reduce the data time points and save up computational power [30,33,78]. Also, downsampling can free up memories due to lesser time points, making this method portable and less costly to implement. The downsampled signal, $x_{i}\left[n\right]$, which is obtained from $d_{i}\left[n\right]$ in Section 2.1, is defined as [79]:

(2) $$x_{i}\left[n\right]=d_{i}\left[Dn\right]=d_{i}\left[10n\right],$$

where *D* is the downsampling factor. The downsampling works by decimating the signal by *D*; that is, keeping only every *D*-th sample and discard the rest.

The resultant signal next has undergone a visual inspection of artifacts. Segments that contained artifacts were removed from the recording. Then, the first 60 s of data are eliminated since they are frequently corrupted by artifacts. Also, at the initial phase of recording, subjects are generally not comfortable yet. From the literature, most of the study used 60 s of recordings, indicating that 60 s of recording is enough to give reliable diagnosis outcomes using qEEG features [29,80]. Furthermore, the establishment of more discriminating characteristics of EEG appears at the beginning part of the recording [81]. Therefore, the next 60 s of the recording is extracted from the recording. Input to the proposed LSTM ECOC-SVM architecture is a 63 × 6000 matrix, representing 60 s of pre-processed EEG recording (i.e., one second of recording is equal to 100 data points). For each time step (i.e., one second), 100 data points are passed to the LSTM. The LSTM is trained using the input EEG for 60-time steps (i.e., 60 s of recording).

### 2.3. Overview of Proposed LSTM ECOC-SVM Architecture

**Figure 1:**
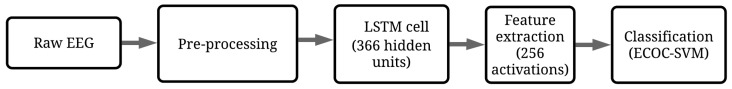
Proposed long short term memory (LSTM) error-correcting output coding support vector machine (ECOC-SVM) architecture.

The proposed LSTM ECOC-SVM architecture inherited the name from both LSTM and ECOC-SVM. The architecture is divided into two parts. LSTM is used to perform feature extraction, while the activations from the LSTM cell (i.e., learnable parameters) are used as features to train an ECOC-SVM to perform classification of non-severe TBI and healthy subject. The overall architecture is presented in Figure 1.

Input to the proposed LSTM ECOC-SVM architecture is a 63 × 6000 matrix, which represents 60 s of pre-processed EEG recording (i.e., one second of recording is equal to 100 data points). The raw EEG signal was pre-processed using the procedure explained in Section 2.2. For each time step (i.e., one second), 100 data points were passed to the LSTM. The LSTM was trained using the input EEG for 60-time steps (i.e., 60 s of recording). The LSTM is set to have 256 hidden units, which will output a feature vectors with 256 values. The output from the LSTM cell is used as features to train an ECOC-SVM to perform classification. Error-correcting output coding (ECOC) is often used together with SVM to perform multiclass classification, as SVM alone can only perform binary classification. ECOC classification needs a coding system to regulate the learners’ training categories (i.e., SVM), and a decoding method that regulates the aggregation of the final prediction for all the binary classifiers. The coding design used in this study is a one-versus-one scheme, also known as an exhaustive matrix scheme. The coding design is shown in Table 1. Value 1 is the notation for positive class, value –1 is for negative class, and value 0 is for ignoring the class. For example, SVM 1 treats the healthy subject as the positive class, mild TBI subject as the negative class, whereas moderate TBI class is omitted. The other SVMs are trained similarly.

When making a prediction, each classifier outputs a “0” or “1”, creating an output code vector. This output vector is compared to each codeword in the matrix, and the class whose codeword has the nearest distance to the output vector is chosen as the predicted class. The process of merging the outputs of individual binary classifiers is known called decoding. Hamming distance is used as the decoding method in this study to look for the minimum distance between the prediction vector and code words, which counts the number of bits that differ. Therefore, the LSTM cell acts as a feature extraction mechanism for the proposed LSTM ECOC-SVM architecture, where the ECOC-SVM acts as the classification mechanism.

Five parameters are fixed for the proposed LSTM ECOC-SVM architecture. Table 2 presents the parameters and their respective value. A learning rate of 0.001 was obtained by conducting extensive experiments, followed by a mini-batch size of 4. $L_{2}$ regularization is set to 0.0005 to prevent overfitting. Overfitting occurs when the learnable weights in the network grow too large to handle the specificity of the examples seen in the training data. Regularization reduces overfitting by penalizing large weights, encouraging smaller weights for the model. In a way, regularization tune the learning of architecture to encourage small weights usage. For the learning of the LSTM via back-propagation, ADAM is selected as the optimizer. The training repetitions per epoch is set to 30 iterations. The training iteration is selected at a moderate value. The reason is to prevent overfitting the network with a higher iteration of training. On the other hand, an iteration that is too less can underfit the network with training data due to insufficient training repetitions.

### 2.4. Training Procedure and Performance Measure

A small dataset usually becomes a challenge in bioinformatics researches due to unexpected constraints, such as the restricted number of patients. One of the common solutions for small dataset issues is utilizing data augmentation as used in image classification research. Unfortunately, this approach is not suitable for mild or moderate TBI patient’s EEG because modifications introduced by the augmentation process, such as the addition of the random noise, can amplify the classification error.

In assessing the designed architecture, the bootstrap approach [82] has been selected to be applied in this research as a solution to overcome the small dataset issues. This resampling method creates bootstrap sample sets in three steps. In the first step, the method will randomly choose the data from the original dataset. Then, the random sample will be combined with the new dataset. In the third step, this combined data will be returned to the original dataset. The first two steps will be reiterated until the generated bootstrap sample set achieves the predefined numbers of samples. It is worth noting that the bootstrap sample set created for the machine learning algorithm will be the amounts of data on the original dataset [83]. A few samples are indeed represented repeatedly, while others are not evaluated at all [83]. Bootstrapping is a helpful tool because the prediction outcomes from the model of trained machine learning utilizing sample sets of bootstrap always present a Gaussian distribution. Besides, 95% confidence interval (CI) can be analyzed to determine the accuracy and stability of the machine learning algorithm from the predictive results.

Efron [82] suggested that 250 iterations can give useful percentile intervals. Therefore, for the proposed architecture design, 250 iterations of the resampled bootstrap sample set are used. To achieve an even ambitious measure of confidence intervals, Efron suggested a minimum of 1000 iterations of resampled bootstrap sample set [82]. Thus, 2000 iterations of bootstrap resampling are performed in the assessment of the final developed architecture. On every bootstrap sample, 3-fold cross-validation is conducted. From the cross-validation, four quantitative evaluations are recorded for each generated bootstrap sample set (i.e., accuracy, sensitivity, specificity, and precision). Ninety-five% CI, mean and standard deviation (SD) are then determined from the documented evaluations.

## 3. Results

Investigations have been done using a simple hill-climbing approach to determine the ideal architecture and setting for the proposed architecture. The search stopped when the performance shows a downtrend, and the parameter with the best performance is selected. Five experiments were conducted to design the proposed LSTM ECOC-SVM architecture. The dataset used in the experiments was discussed in Section 2.1. Furthermore, the training procedures and performance measures used were presented in Section 2.4.

Each of the experiments is explained in six sections. Section 3.1 presents the experiments in determining the best learning rate for the proposed architecture. Subsequently, Section 3.2 discussed the experiments to choose the optimum mini-batch size and analysis of the results. In the next section (i.e., Section 3.3), experiments are conducted to determine the optimum number of hidden units for the LSTM cell. This is followed by Section 3.4, which presenting the experiments to determine the best optimizer for the learning of LSTM. The next section (i.e., Section 3.5) presents the evaluation and final touch up for the proposed LSTM ECOC-SVM architecture. The effects of pre-processing on the proposed architecture are also explored in the latter part of this section. Finally, in Section 3.6, the proposed architecture was compared to similar works in the literature, as well as our previous studies.

### 3.1. Selection of Optimum Learning Rate

LSTM is a machine learning approach that learn via backpropagation to determine the learnable weight and bias for respective gates in the LSTM cell (i.e., forget gate (*f*), input gate (*i*), cell candidate gate (*s*) and output gate (*o*). Therefore, the learning rate is one important parameter to determine the learnable parameter update of the architecture in conjunction with the gradient descent. If the learning rate value is set too high, although it can cause the architecture to converge rapidly, the architecture may be reached to a sub-optimal point, which may not give the maximum potential to the architecture. Besides, the loss function will overshoot the minimum error point, causing oscillation between the gradient descent.

Conversely, applying an extremely small learning rate will mostly result in longer training time to converge. Besides, it can also cause the training to be stuck at a point after all the training repetitions are done. Therefore, a good learning rate has to be determined to ensure effective learning of the architecture. The present research suggests that an effective learning rate can be approximated by starting with a larger value and decreasing it at every repetition, with a learning rate of 0.1 being a good starting point [84]. An initial LSTM (i.e., with the setting of one LSTM cell with 64 hidden units, one FC layer with three neurons, mini-batch size of eight, and ADAM optimizer) was used to conduct the experiments. The learning rates explored are 0.1, 0.01, 0.001, and 0.0001 respectively. Table 3 presents the performance of each learning rate.

By decreasing the learning rate from 0.1 to 0.001, there is an improvement of 6.16% of classification accuracy (i.e., improves from 64.97% to 71.13%). Further decrements of the learning rate do not improve the architecture’s performance but worsened it (i.e., degraded from 71.13% to 67.11% in terms of classification). Referring to Table 3, a learning rate of 0.001 gives the highest outcome in all performance measures, with the classification accuracy of 71.13%, the sensitivity of 70.30%, the specificity of 85.87% and the precision of 73.84%. At this learning rate, the step is optimum to search for the best learnable parameters of the architecture, compared to other learning rate values.

Also, the result indicated a high learning rate of 0.1 caused overstepping of the learnable parameters update, thus missing out on the optimum local minimal. The step taken to update the parameters over-shoot and the training may neither converge nor diverge. Weight and bias changes can be too big, causing the optimizer to miss out on the local minimal and worsen the training loss.

Reducing the learning rate to 0.01 can improve the performance, whereas 0.001 is the threshold point. Learning rates that are smaller than 0.001 do not further improve the performance of the architecture. By using a lower learning rate can cause the architecture to take a longer time to optimize because the steps taken towards the minimum of the loss function are small. Hence, more epoch repetitions are needed to reach the local optimum, resulting in longer training time. By tolerating some learning time, a learning rate of 0.001 is selected as the optimum value for the proposed architecture.

### 3.2. Selection of Optimum Mini Batch Size

Deep learning such as CNN uses backpropagation for learnable parameters update. LSTM cell learns the same way through backpropagation. The entire training dataset was divided into a smaller subset (i.e., known as mini-batch) and supplied to the LSTM to update the learnable parameters. Therefore, the optimum mini-batch size must be obtained as it affects the quality of the learning of the architecture.

A large mini-batch size causes a higher computational power. Besides, an overly large mini-batch size will result in performance deterioration of the architecture as it will result in a huge step of learnable parameter update, resulting in converging to a sharp local-minimum [85]. On the other hand, small mini-batch sizes result in a noisier update as more changes are done for the learnable parameters. Hence, smaller mini-batch size offers a regularization effect and lower generalization error. It is also worth to mention that a smaller mini-batch size requires a lower computational power. Thus, it is important to determine an optimum mini-batch size to allow the LSTM to converge better and more stable.

A 32 mini-batch size was the recommended default value by several studies [84,86]. In this study, the epoch size of the input EEG time series is 36 (i.e., there are a total of 36 EEG recordings). Therefore, each mini-batch size represents the number of EEG recordings supplied to the architecture each pass. Experiments are conducted using the mini-batch size of 1, 2, 4, 8, 16, 32, 64 on the architecture of one LSTM cell with 64 hidden units, one FC layer with three neurons, ADAM optimizer, and a learning rate of 0.001 determined via experiments conducted in Section 3.1. Performance of each mini-batch size is tabulated in Table 4.

From Table 4, it can be seen that when the mini-batch size increases from 1 to 4 (i.e., the number of EEG recording in one pass increases), the performance of the architecture improves gradually in all performance measures. LSTM architecture tends to learn more effectively when there are more EEG time series supplied to it. However, the performance of the architecture worsens when mini-batch sizes of 8 and 16 were used (i.e., classification decreased from 71.99% to 61.34%). Nevertheless, mini-batch sizes of 32 and 64 present a small bounce back in performance, archiving the classification accuracy of 70.28% and 70.93%, respectively.

From the trend of the results, it was shown that a mini-batch size of 4 gives the best performance, achieving the classification accuracy of 71.99%, the sensitivity of 70.03%, the specificity of 86.25% and the precision of 73.12%. The mini-batch size of 4 can efficiently generalize the EEG time series and converge to a flat minimal, giving the architecture a better generalization of trained data.

On the other hand, the result also provides an insight that a mini-batch size larger than 4 caused inefficient training of the LSTM. This results in a sharp local-minimum convergence, which is not ideal in backpropagation. Moreover, mini-batch sizes of 32 and 64 passed the whole dataset at once to the LSTM. They require a high computation power, and at the same time, the full batch gradient trajectory can result in non-quality learnable parameters update (i.e., bad optimum point landing). Optimum mini-batch size has to be obtained, so the backpropagation injects enough noise to each gradient update while achieving an effective and speedy convergence to the local-minimum.

The results show that a relatively smaller mini-batch size carries better generalization ability. Using fewer examples can result in a less accurate estimate of the error gradient that is highly dependent on the training samples. Hence, it results in a noisy estimate, and in return, caused noisy updates to the model weights (i.e., updates with estimates of the error gradient that varies from each other). Nevertheless, these noisy updates can result in faster learning and developed a more robust model.

### 3.3. Selection of Optimum Hidden Units

The hidden units in an LSTM correspond to the dimension of information learned from previous time steps, regardless of the sequence length of the supplied time series. It is also known as the hidden size, which carries the same definition of the number of hidden nodes for ANN. The number of hidden units has to be selected carefully. Overfitting of the training data will take place if the number of the hidden unit is too large.

Experiments were conducted using 8, 16, 32, 64, 128, and 256 hidden units. There are no clear guidelines on determining the suitable number of the hidden unit; hence it has to be determined empirically. These experiments were conducted using an LSTM architecture of one LSTM cell, one FC layer with three neurons, an ADAM optimizer, a learning rate of 0.001, and a mini-batch size of 4, where the learning rate and mini-batch size are determined from previous experiments (i.e., Section 3.1 and Section 3.2). The results from the experiments were shown in Table 5.

From the results, eight hidden units present the lowest performance, with the classification accuracy of 69.34%, the sensitivity of 66.60%, the specificity of 82.25%, and the precision of 71.96%. A smaller number of hidden units of the LSTM cell cause the gates to have low learning ability and results in underfitting, as there are fewer hidden units to fit in the features. The lower number of hidden units has failed to detect and learn from the activations of the LSTM cell.

Subsequently, the classification accuracy of the LSTM architecture improves when the number of the hidden units is increased to 64 (i.e., improves from 69.34% to 71.99%). There is a small degradation of performance in the architecture with 128 hidden units but bounced to 72.09% of classification accuracy when 256 hidden units are used. The experiments are not further conducted for 512 hidden units due to computational power restrictions. Thus, 256 hidden units are the peak performance among all variations. In this case, 256 hidden units have sufficient capacity to fit in the amount of information supplied by the EEG time series, avoiding the risk of underfitting and overfitting. Optimally, a balance is met where there is an equal capacity of hidden units to learn from all the information from the input time series.

### 3.4. Selection of Optimizer for Backpropagation

LSTM carries learnable parameters (i.e., weight and bias) that updates via backpropagation. The goal of the backpropagation learning is to minimize the difference between the predicted output and the actual result (i.e., the error). For the update of LSTM’s learnable parameters, the time series of EEG was forward passed per time step. The cross-entropy loss function is used to compute the error (i.e., the difference between predicted result and actual result). In this study, the loss function that is used computed the error is cross-entropy. A study has been done, and it is shown that cross-entropy performs better than the usual mean squared error (MSE) loss function [87]. In the initial forward pass of a CNN architecture, weights in the hidden layers are arbitrary. The optimum weights have to be calculated by an optimizer based on the output of the loss function. An optimizer improves the performance of the architecture by minimizing the error. As the learnable parameters were updated at every time steps, the learning process was known as backpropagation-through-time (BPTT).

In the initial forward pass, the learnable parameters are arbitrary. An optimizer has to be used to calculate the optimum learnable parameters based on the output of the loss function. The quality of the LSTM corresponds to the ability of the optimizer to minimize the error. Two optimizers were evaluated in this study (i.e., SGD and ADAM). The architecture used to evaluate the optimizers is made up of one FC layer with three neurons, a learning rate of 0.001, 256 hidden units, and a mini-batch size of 4, where the learning rate, mini-batch size, and the number of hidden units are determined from previous experiments (i.e., Section 3.1, Section 3.2 and Section 3.3).

The performance for each of the optimizer is presented in Table 6. The results showed that architecture using ADAM performs better than SGD. Also, both of the optimizers shown stable performance by presenting a low standard deviation (i.e., below 9). SGD with momentum presents a comparable performance (i.e., the classification accuracy of 70.87%, the sensitivity of 71.27%, the specificity of 85.87%, and the precision 74.50%).

It shows that SGD with momentum is a good option as it provides momentum towards the correct direction of gradient descent for the local-minimum. The original SGD without momentum oscillate along the path of steepest descent towards the optimum, making the architecture harder to final the local minima. Adding a momentum term to the weights update can overcome this issue by adding momentum in the direction of consistent gradients and discard the momentum if gradients are in opposite directions [88]. SGD with momentum shows comparable performance and converges faster than the original SGD as bigger steps are taken towards the same direction following the momentum.

However, this experiment is targeted to look for the best-performed optimizer. Using the same architecture, ADAM can provide higher performance, hitting classification accuracy of 72.09%. ADAM is an optimizer that is a combination of SGD with momentum and root mean square propagation (RMSProp). Therefore, ADAM carries the advantage of momentum, which solves the problem of random oscillation and also the strong side of RMSProp that changes the step size by adapting to the gradient.

There is always an on-going argument in the comparison between SGD with momentum and ADAM, in which some studies stated SGD with momentum is a better optimizer [89]. Despite that, this experiment shows that ADAM is a better optimizer compared to SGD in classifying non-severe TBI and healthy subjects. Therefore, it can be presumed that the option of optimizer varies for different problem-solving.

Based on the result in Table 6, ADAM is well performed by computing a unique learning rate for each of the learnable parameters, which is more compatible with the classification objective of this study. The different learning rate is assigned to the update of each weight, and bias enhanced the learning of the architecture by avoiding inappropriate steps that deviate away from the local optimum.

By solving random oscillation of the local-minimum search, ADAM converges well to the local-minimum and present a high performance with the classification accuracy of 72.09%, the sensitivity of 70.07%, the specificity of 86.70%, and the precision of 74.93%. In conclusion, ADAM is selected as the optimizer for the proposed LSTM architecture.

### 3.5. Construction of Proposed Architecture

From all the experiments conducted in previous sections, the LSTM architecture with the optimized parameters was obtained (i.e., one LSTM cell with 256 hidden units, 0.001 learning rate, mini-batch size of 4, and ADAM as optimizer). The architecture can present a comparable performance with the classification accuracy of 72.09%, the sensitivity of 70.07%, the specificity of 86.70%, and the precision of 74.93%. However, the architecture has to be improved for better performance. The performance suggested that the SoftMax classifier at the output of the last FC layer did not perform well enough in classification. Hence, it becomes a motivation to propose architectures to replace the SoftMax.

In this study, a multiclass classification has to be performed. Thus, the error-correcting output coding (ECOC) algorithm is introduced to combined with SVM. SVM is a robust and powerful binary classifier due to its ability to perform class separation and the facilities of the kernel space. Combining SVM with the ECOC algorithm can handle the multiclass problem efficiently by utilizing the binary set of ECOC with suitable coding rules to achieve a non-linear classification while reducing the bias and variance of the trained models. There are other choices of machine learning methods that can perform multiclass classification by itself without any coding rules. However, a study has been conducted to show ECOC-SVM outperforms them [90]. Hence, it became our choice to evaluate its potential to replace SoftMax. In this section, experiments are conducted by using the obtained LSTM architecture, and Softmax is replaced by ECOC-SVM. Activations from the hidden units of the LSTM cell are used as features to train an ECOC-SVM. The performance of the LSTM ECOC-SVM is tabulated in Table 7 together with the LSTM that uses Softmax as the classifier.

From Table 7, it is shown that ECOC-SVM that are trained by the activations from the LSTM cells outperformed the LSTM with SoftMax classifier, presenting the classification accuracy of 98.09%, the sensitivity of 98.50%, the specificity of 98.87% and the precision of 97.86%. There was a drastic improvement of 26% in terms of the classification accuracy. Compared to SoftMax, ECOC-SVM is more powerful and robust in performing class separation. To perform multiclass classification, ECOC utilizes the coding rules and binary SVM, creating a well-performed architecture. SVM can give a prediction towards the local objective, providing distinct scores for the predicted EEG, where the detail of individual scores does not take a count on the final prediction. On the other hand, SoftMax computes probabilities for each of the classes. Non-related components (i.e., noise and artifacts) can cause the decision boundaries to vary as it will recalculate and include the influence of the non-related components. This becomes a disadvantage in architecture that solve classification problems involving EEG signal as noise and artifacts in the signals were unavoidable. Therefore, ECOC-SVM is a better option than SoftMax.

Upon this stage of study, the EEG time series supplied to the previous experiments (i.e., Section 3.1, Section 3.2, Section 3.3 and Section 3.4) did not undergo any pre-processing. LSTM is a time-dependency architecture where the correlation of each time step is stored in the hidden units (i.e., LSTM cells). Therefore, any noises and artifacts can directly impact the quality of the architecture training as noises and artifacts can be remembered from the previous time step. To evaluate the effect of the pre-processing, in this section, the EEG time series have undergone a pre-processing procedure described in Section 2.2 and used to train the proposed LSTM ECOC-SVM architecture. Its performance was presented in Table 8 together with the same architecture trained using raw EEG.

From Table 8, using pre-processed EEG improved the proposed LSTM ECOC-SVM architecture from 98.04% to 100% in term of the classification accuracy. Besides, the proposed LSTM ECOC-SVM presents the best performance by achieving 100% in all the performance measures. Also, the standard deviation of 0 indicates that the proposed LSTM ECOC-SVM architecture has a very stable performance throughout the 250 bootstrap resampling run and cross-validation. The pre-processing procedure used is efficient in removing noises and artifacts in the EEG time series, providing precise information throughout the training and BPTT without being confused by unwanted elements (i.e., noises and artifacts). This results in an effective learnable parameter update through time, where each cell is well trained with the ability to remember important information from the previous time step and avoided overfitting by discarding unrelated information.

The experiment is repeated using 2000 bootstrap resampling to ensure the high performance of the proposed LSTM ECOC-SVM architecture using the pre-processed EEG signal. Its performance was tabulated in Table 9, together with the experiment done using 250 bootstrap resampling. It was shown that the experiment done with 2000 bootstrap resampling performed the same with the one done using 250. Again, the result assured that the proposed LSTM ECOC-SVM could classify non-severe TBI and healthy subjects accurately and precisely with 100% of classification accuracy, sensitivity, specificity, and precision.

### 3.6. Assessment of the Proposed Method with Existing Works

Currently, there is no available work which classifies non-severe TBI and healthy group. Thus, the performance of the proposed method is only assessed with four similar methods. The first comparison method is the work by Brink et al. [30] that utilized Naive Bayes to classify TBI from task-free EEG. The second method for comparison classifies the EEG signal by employing the AdaBoost classifier and is developed by McNerney et al. [29]. The third and fourth methods were our previously developed methods based on SVM [91,92]. In our previous work, the same pre-processing procedure presented in Section 2.2 was used to pre-process the data. Alpha band power and theta power spectral density (PSD) were extracted to train two SVM classifiers, respectively. For a reasonable assessment, the same dataset and training process is utilized. The performance of each method and the proposed LSTM ECOC-SVM is shown in Table 10.

Asserting that the extracted features from the frequency bands can provide valuable data to the classifier, the four comparison methods [29,30,91,92] used the frequency band-based features. In contrast, the proposed approach in this research does not require any extraction of the features. The EEG is passed into the proposed architecture per time step, where important information from each time step is remembered by the gates of the LSTM cell. In a way, the correlation between each time step is extracted using BPTT and stored as activations. The proposed architecture fully utilized the temporal advantage of the EEG time series. By avoiding extensive feature extraction, the proposed architecture can directly learn effectively from the pre-processed EEG signal.

Results have shown the proposed architecture outperformed the other two methods with high performance with the classification accuracy of 100%, the sensitivity of 100%, the specificity of 100%, and the precision of 100%. Naive Bayes presented a comparable performance (i.e., the classification accuracy of 97.01%). However, to ensure such high performance, pre-processing and feature extraction has to be performed in care to ensure high quality and discriminative features can be extracted. On the other hand, the AdaBoost classifier is only able to present a classification accuracy of 62.68%.

Naive Bayes ignored the dependence of the EEG channels and assumed that each feature does not correlate to each other. This may cause a loss in information during the classifier training process because correlations of the channels have been neglected. As such, the proposed approach which uses LSTM should resolve the limitation of Naive Bayes by taking into account the correlation between time steps and also between channels. On the other hand, although the AdaBoost classifier needs less parameter tuning and is simple to use, it is prone to outliers and noise, which is inevitable in EEG signals. Thus, more effort must be taken to ensure the noise and artifacts are fully eliminated for the successful training of classifiers. The proposed method only has to undergo simple bandpass filtering and to remove segments containing artifacts yet with a performance of 100% in all measures.

Alpha band power and theta band spectral density (PSD) were extracted from the EEG to become the SVM training features for our previous works [91,92]. As expected, they have a lower classification performance as compared with the proposed method (i.e., LSTM ECOC-SVM). This is because the information from alpha band power and theta PSD are not adequate to classify non-severe TBI resting-state EEG signals. More information is needed to sufficiently train an SVM, such as correlation coefficient, phase difference, and others.

## 4. Conclusions

In this paper, experiments were conducted to obtain the optimum learning rate, mini-batch size, number of hidden units, and optimizer. Optimum parameters determined included the learning rate of 0.001, the mini-batch size of 4, 256 hidden units, and the ADAM optimizer. The proposed LSTM ECOC-SVM architecture is made up of one LSTM cell with 256 hidden units and an ECOC-SVM classifier. It was shown that the pre-processed EEG signal could supply quality information to the proposed architecture, improving its performance compared to the one trained using raw EEG signal. By fully utilizing the temporal advantage of EEG, the proposed architecture present a 100% high performance of classification accuracy, sensitivity, specificity, and precision. The proposed method has substantially outperformed similar works in the literature, as well as in our previous studies. 

## Figures and Tables

**Table 1:** Error-correcting output coding (ECOC) SVM coding design.

	SVM 1	SVM 2	SVM 3
Healthy	1	1	0
Mild	–1	0	1
Moderate 3	0	–1	–1

**Table 2:** Parameters and values.

Parameter	Setting
Learning rate	0.001
Mini-batch size	4
$L_{2}$ regularization	0.0005
Optimizer	ADAM
Training repetitions per epoch	30

**Table 3:** Accuracy, Sensitivity, Specificity and Precision for Various Learning Rate Using LSTM. (The Numbers in Bold Indicate the Best Value Obtained for Each Quality Measure).

Learning	Accuracy ± SD	Sensitivity ± SD	Specificity ± SD	Precision ± SD
Rate	[CI]	[CI]	[CI]	[CI]
0.1	64.97 ± 9.49	62.67 ± 15.18	83.38 ± 8.17	69.00 ± 18.12
	[63.79 66.15]	[60.78 64.56]	[83.37 85.40]	[66.74 71.26]
0.01	69.07 ± 9.11	66.37 ± 15.51	87.05 ± 7.37	73.75 ± 18.68
	[67.93 70.20]	[64.43 68.30]	[86.13 87.97]	[71.42 76.08]
0.001	**71.13 ± 8.65**	**70.30 ± 14.13**	**85.87 ± 7.05**	**73.84 ± 15.63**
	**[70.06 72.21]**	**[68.54 72.06]**	**[84.99 86.75]**	**[71.89 75.79]**
0.0001	67.11 ± 8.90	64.30 ± 15.02	84.85 ± 7.76	71.09 ± 14.50
	[66.00 68.22]	[62.43 66.17]	[83.88 85.82]	[69.29 72.90]

**Table 4:** Accuracy, Sensitivity, Specificity and Precision for Various Mini Batch Size Using LSTM. (The Numbers in Bold Indicate the Best Value Obtained for Each Quality Measure).

Mini	Accuracy ± SD	Sensitivity ± SD	Specificity ± SD	Precision ± SD
Batch Size	[CI]	[CI]	[CI]	[CI]
1	69.84 ± 8.76	67.27 ± 15.24	85.57 ± 8.17	72.37 ± 17.47
	[68.75 70.94]	[65.37 69.16]	[84.55 86.58]	[70.19 74.54]
2	71.59 ± 8.75	69.40 ± 15.55	86.53 ± 7.26	73.71 ± 17.69
	[70.50 72.68]	[67.46 71.34]	[85.63 87.44]	[71.50 75.91]
4	**71.99 ± 8.68**	**70.03 ± 13.99**	**86.25 ± 8.12**	**73.12 ± 19.12**
	**[70.91 73.07]**	**[68.29 71.78]**	**[85.24 87.26]**	**[70.74 75.50]**
8	71.13 ± 8.65	70.30 ± 14.13	85.87 ± 7.05	73.84 ± 15.63
	[70.06 72.21]	[68.54 72.06]	[84.99 86.75]	[71.89 75.79]
16	61.34 ± 8.36	61.57 ± 15.37	80.17 ± 10.12	62.16 ± 20.75
	[60.30 62.39]	[59.65 63.48]	[78.91 81.43]	[59.57 64.74]
32	70.28 ± 8.14	68.17 ± 14.57	85.62 ± 8.29	72.77 ± 17.14
	[69.26 71.29]	[66.35 69.98]	[84.58 86.65]	[70.64 74.91]
64	70.93 ± 8.61	69.87 ± 15.50	85.63 ± 7.27	72.71 ± 17.42
	[69.86 72.01]	[67.94 71.80]	[84.73 86.54]	[70.54 74.88]

**Table 5:** Accuracy, Sensitivity, Specificity and Precision for Various Number of Hidden Unit Using LSTM. (The Numbers in Bold Indicate the Best Value Obtained for Each Quality Measure).

No. of	Accuracy ± SD	Sensitivity ± SD	Specificity ± SD	Precision ± SD
Hidden Unit	[CI]	[CI]	[CI]	[CI]
8	69.34 ± 8.35	66.60 ± 14.77	82.25 ± 7.29	71.96 ± 16.41
	[68.30 70.38]	[64.76 68.44]	[84.34 86.16]	[69.92 74.00]
16	69.91 ± 8.55	68.67 ± 15.14	84.77 ± 7.85	71.87 ± 16.02
	[68.85 70.98]	[66.78 70.55]	[83.79 85.74]	[69.88 73.87]
32	70.57 ± 8.88	69.50 ± 15.51	85.23 ± 7.64	72.08 ± 16.68
	[69.46 71.67]	[67.57 71.43]	[84.28 86.19]	[70.00 74.15]
64	71.99 ± 8.68	70.03 ± 13.99	86.25 ± 8.12	73.12 ± 19.12
	[70.91 73.07]	[68.29 71.78]	[85.24 87.26]	[70.74 75.50]
128	71.81 ± 8.13	70.17 ± 14.30	86.28 ± 7.97	74.63 ± 16.00
	[70.80 72.82]	[68.39 71.95]	[85.29 87.28]	[72.64 76.63]
256	**72.09 ± 8.71**	**70.07 ± 15.07**	**86.70 ± 7.94**	**74.93 ± 17.06**
	**[71.00 73.17]**	**[58.19 71.94]**	**[85.71 87.69]**	**[72.80 77.05]**

**Table 6:** Accuracy, Sensitivity, Specificity and Precision for Different Optimizer Using LSTM. (The Numbers in Bold Indicate the Best Value Obtained for Each Quality Measure).

Type of Optimizer	SGD	ADAM
Accuracy ± SD [CI]	70.87 ± 8.33 [69.83 71.90]	**72.09 ± 8.71 [71.00 73.17]**
Sensitivity ± SD [CI]	71.27 ± 13.67 [69.56 72.97]	**70.07 ± 15.07 [58.19 71.94]**
Specificity ± SD [CI]	85.87 ± 7.12 [84.98 86.75]	**86.70 ± 7.94 [85.71 87.69]**
Precision ± SD [CI]	74.50 ± 13.88 [72.77 76.23]	**74.93 ± 17.06 [72.80 77.05]**

**Table 7:** Accuracy, Sensitivity, Specificity and Precision for Different Classifier Using LSTM. (The Numbers in Bold Indicate the Best Value Obtained for Each Quality Measure).

Classifier	SoftMax	ECOC-SVM
Accuracy ± SD [CI]	72.09 ± 8.71 [71.00 73.17]	**98.09 ± 2.10 [97.83 98.35]**
Sensitivity ± SD [CI]	70.07 ± 15.07 [58.19 71.94]	**98.50 ± 3.29 [98.09 98.91]**
Specificity ± SD [CI]	86.70 ± 7.94 [85.71 87.69]	**98.87 ± 2.00 [98.62 99.12]**
Precision ± SD [CI]	74.93 ± 17.06 [72.80 77.05]	**97.86 ± 3.74 [97.40 98.33]**

**Table 8:** Accuracy, Sensitivity, Specificity and Precision for Raw and Pre-processed electroencephalogram (EEG) using LSTM ECOC-SVM. (The Numbers in Bold Indicate the Best Value Obtained for Each Quality Measure).

EEG	Raw	Pre-Processed
Accuracy ± SD [CI]	98.04 ± 2.19 [97.77 98.32]	**100 ± 0 [100 100]**
Sensitivity ± SD [CI]	98.40 ± 3.61 [97.95 98.85]	**100 ± 0 [100 100]**
Specificity ± SD [CI]	98.85 ± 2.27 [98.57 99.13]	**100 ± 0 [100 100]**
Precision ± SD [CI]	97.86 ± 4.11 [97.35 98.38]	**100 ± 0 [100 100]**

**Table 9:** Accuracy, Sensitivity, Specificity and Precision for the Performance of 250 and 2000 Bootstrap Resampling Using LSTM ECOC-SVM with Pre-processed EEG. (The Numbers in Bold Indicate the Best Value Obtained for Each Quality Measure).

EEG	2000	250
Accuracy ± SD [CI]	**100 ± 0 [100 100]**	100 ± 0 [100 100]
Sensitivity ± SD [CI]	**100 ± 0 [100 100]**	100 ± 0 [100 100]
Specificity ± SD [CI]	**100 ± 0 [100 100]**	100 ± 0 [100 100]
Precision ± SD [CI]	**100 ± 0 [100 100]**	100 ± 0 [100 100]

**Table 10:** Accuracy, Sensitivity, Specificity and Precision for the Performance existing works and proposed convolutional neural network (CNN) ECOC-SVM Voting Ensembles Architecture. (The Numbers in Bold Indicate the Best Value Obtained for Each Quality Measure).

Method	Accuracy ± SD	Sensitivity ± SD	Specificity ± SD	Precision ± SD
	[CI]	[CI]	[CI]	[CI]
Naive Bayes [30]	97.01 ± 0.05	99.81 ± 0.23	95.74 ± 0.74	92.15 ± 1.25
	[96.99 97.03]	[99.80 99.82]	[95.70 95.77]	[92.09 92.20]
Adaboost [29]	62.68 ± 9.34	67.67 ± 16.62	82.28 ± 9.62	67.00 ± 13.48
	[62.27 63.09]	[66.94 68.40]	[81.85 82.70]	[66.41 67.59]
SVM(PSD) [91]	63.64 ± 8.42	76.91 ± 13.52	83.11 ± 8.09	70.41 ± 11.53
	[63.27 64.01]	[76.32 77.50]	[82.75 83.46]	[69.91 70.92]
SVM(power) [92]	52.22 ± 8.65	76.24 ± 15.26	66.71 ± 9.93	53.86 ± 9.75
	[51.84 52.60]	[75.57 76.91]	[66.27 67.14]	[53.43 54.29]
LSTM ECOC-SVM	**100 ± 0**	**100 ± 0**	**100 ± 0**	**100 ± 0**
	**[100 100]**	**[100 100]**	**[100 100]**	**[100 100]**
